# Influence of mental health service provision on the perceived quality of life among psychiatric outpatients: associations and mediating factors

**DOI:** 10.3389/fpsyt.2023.1282466

**Published:** 2024-01-16

**Authors:** Lars-Olov Lundqvist, Patrik Rytterström, Mikael Rask, David Brunt, Tabita Sellin, Katarina Grim, Ingrid Rystedt, Agneta Schröder

**Affiliations:** ^1^University Health Care Research Center, Faculty of Medicine and Health, Örebro University, Örebro, Sweden; ^2^Division of Nursing Sciences and Reproductive Health, Department of Health, Medicine and Caring Sciences, Linköping University, Norrköping, Sweden; ^3^School of Health and Caring Sciences, Linnaeus University, Växjö, Sweden; ^4^Department of Social and Psychological Studies, Karlstad University, Karlstad, Sweden; ^5^Division of Society and Health, Department of Health, Medicine and Caring Sciences, Linköping University, Linköping, Sweden; ^6^Department of Nursing, Faculty of Health, Care and Nursing, Norwegian University of Science and Technology (NTNU), Gjövik, Norway

**Keywords:** mediator model, mental health, outpatient psychiatric care, service provision, structural equation modelling, quality of life

## Abstract

**Objective:**

To investigate the relationship between perceived mental health service provision and quality of life (QoL) as perceived by patients in psychiatric outpatient care.

**Methods:**

A total of 373 adult patients registered at 15 psychiatric outpatient clinics in three regions in central and southern Sweden were included in the study. Survey data were collected using a questionnaire on mental health service provision, symptom severity, recovery, clinical diagnosis, sociodemographics (serving as independent variables) and QoL (serving as the dependent variable). Three aspects of mental health service provision were used: patients’ perceived quality of care, perceived staff-patient interaction, and patient reported psychiatric treatments. Structural equation modelling was used to model the relationship among the variables.

**Results:**

Variables in mental health service provision showed few direct associations with patients’ perceived QoL. Instead, the associations of mental health service provision on QoL were mainly mediated through symptom severity and recovery. These relationships were retained after adjusting for sociodemographic variables and clinical diagnoses. The final model achieved excellent goodness of fit (*χ*^2^ = 49.502, *p* = 0.230, RMSEA = 0.020, CFI = 0.997 and a SRMR = 0.024).

**Conclusion:**

This study shows that mental health service provision is associated with patients’ perceived QoL; however, this association is mostly indirect and mediated by reduced symptom severity and increased recovery. This finding can help inform the design of future interventions to enhance service provision to improve patients’ QoL.

## 1 Introduction

Psychiatric outpatient care is designed to offer comprehensive, continuous treatment and support for individuals to cope with mental health conditions to enabling them to sustain their everyday lives within their community (1). A primary goal of the provided care is to improve patients’ quality of life (QoL) (2–6), which encompasses their perceptions of life circumstances within their cultural context, considering their aspirations, expectations, and concerns (7). However, beyond psychiatric clinical symptoms (3, 8–11), various sociodemographic, social, occupational, and financial factors may contribute to patients’ QoL. For instance, studies have indicated that older age (12, 13), female gender (12, 14), relationship problems (11, 12), lower education levels (10, 13, 15), and unemployment (11, 13, 16–18) are associated with lower QoL in patients with mental illness.

However, the multitude of variables linked to QoL makes the research in this area fragmented and challenging to comprehend (19). While attempts have been made to propose models demonstrating the relationships between various variables and QoL (20–22), explicit models that effectively guide the understanding of how mental health service provision impacts QoL are still lacking. The relationship between mental health service provision and patients’ perceived QoL is intricate and may not be adequately explained solely through simple correlations. Employing more complex modelling techniques, such as mediation analysis (23, 24), can help elucidate the complex relationships, where an intermediate (mediating) variable or factor may clarify the connection between the quality of mental health services and patients’ perceived QoL.

Upon reviewing existing literature, it becomes evident that while numerous factors influence QoL, only a selected few have been thoroughly investigated as potential mediators impacting QoL. For instance, studies have delved into variables such as recovery (21, 25) and symptom severity (22, 25–27) but have yet to explore many others in depth. Within the scope of recovery, notable components encompass empowerment, agency, and hope (21, 25, 28). This emphasis on recovery has spurred a growing body of research over the past three decades, prompting mental health systems globally to advocate for recovery-oriented services. These services encourage active involvement and choice for individuals seeking treatment and support (29).

While the concept of recovery from mental illness traditionally involved symptom absence and restoration to a pre-illness state, the contemporary viewpoint extends beyond this notion. Modern perspectives emphasize personal growth and development, surpassing the adverse effects of mental illness to establish a fulfilling and meaningful life (30). Therefore, recovery encompasses more than mere symptom remission or a return to prior functioning levels. Interestingly, a meta-analysis of 20 articles highlights that individuals with schizophrenia can experience personal recovery despite persisting symptoms of psychosis (31). Consequently, recovery and symptom severity may function as partially independent markers of mental health status.

**Figure 1:**
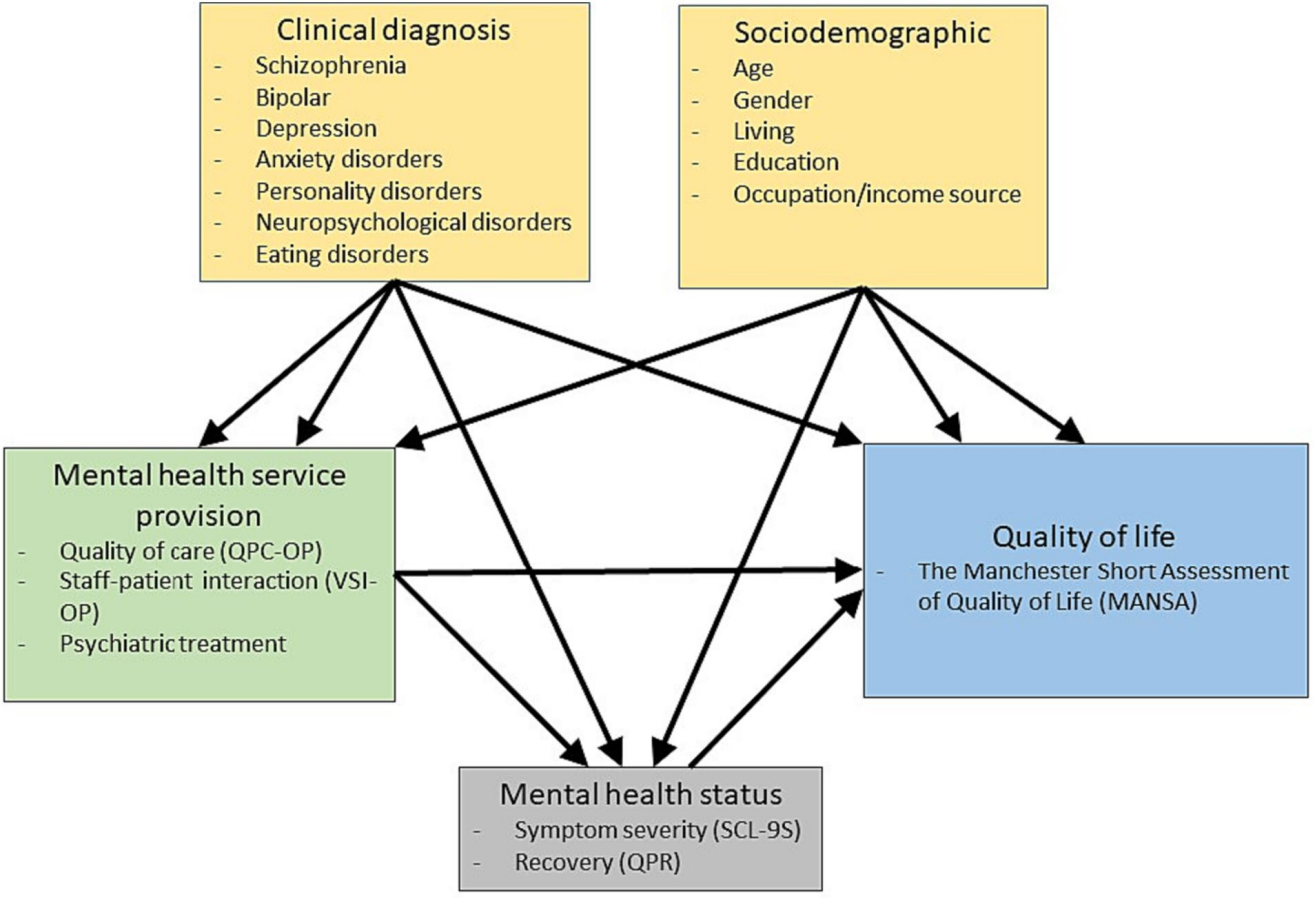
Conceptual model of mental healthcare service provision impact on patients’ perception on quality of life.

Building upon the findings of prior research, a conceptual model (Figure 1) has been proposed. According to this model, variables within mental health services can directly influence QoL or operate via mental health status variables that directly impact QoL. Additionally, sociodemographic variables directly influence QoL alongside patients’ clinical diagnoses.

### 1.1 Aim

The aim is to examine the relationship between perceived mental health service provision and QoL among patients in psychiatric outpatient care.

## 2 Methods

### 2.1 Participants and procedure

The sample used in this study originated from 15 of totally 17 psychiatric outpatient clinics in three regions in central and southern Sweden (Region Kronoberg, Region Värmland, and Region Örebro län) including approximately 795,000 inhabitants. The clinics serve both the urban and the rural population. They are staffed by multi-professional teams. Patients with different mental illnesses can be admitted to the clinic by self-referral or by referral from other caregivers. A questionnaire containing five standardised instruments (described below) and questions concerning sociodemographic characteristics, diagnoses, and psychiatric treatments was distributed to the 15 outpatient clinics. Inclusion criteria for participants encompassed being 18 years of age or older, being able to understand and read Swedish, and cognitively able to answer the questionnaire. Patients eligible for participation were informed orally and in writing by a designated member of staff, who also ensured that the patients were able to answer the questionnaire in a valid way. Those who gave oral consent were asked to complete the questionnaire anonymously prior to leaving the clinic. A total of 706 questionnaires were returned; however, 333 questionnaires were discarded due to 15% or more missing items in any of the instruments included in the questionnaire. The final sample thus comprised 373 patient questionnaires. The patients were between 18 and 87 years old with a mean age of approximately 35 years. The majority of patients were women, had upper secondary education but only one in five patients was working. Characteristics of the study participants are shown in Table 1.

**Table 1:** Participant characteristics (*N* = 373).

Category	Variable	Frequency	%
Sociodemographic			
	Age (years)	35.1	12.87
	Gender		
	Woman	268	72%
	Man	105	28%
	Living		
	Alone	201	54%
	With partner	172	46%
	Education		
	Did not complete school	10	3%
	Compulsory school	59	16%
	Upper secondary school	234	63%
	Higher education	70	24%
	Occupation/income source		
	Work	86	23%
	Unemployed	25	7%
	Sick pay	118	32%
	Sickness compensation^b^	45	12%
	Activity compensation for reduced work capacity^c^	34	9%
	Retirement pension^d^	7	2%
	Student	33	9%
	Other	25	7%
Clinical diagnosis^a^			
	Anxiety disorders	110	30%
	Bipolar	39	11%
	Dependency disorder	2	1%
	Depression	83	22%
	Eating disorders	20	5%
	Neuropsychological disorders	90	24%
	Personality disorders	43	12%
	Schizophrenia	28	8%
	No diagnosis reported	90	24%
Mental health provision			
Treatment^a^			
	Pharmacological	306	82%
	ECT	25	7%
	Counselling	256	69%
	Psychotherapy	176	47%
	Other	78	21%
Previous visits			
	First time	11	3%
	1 time	13	4%
	2–5 times	57	15%
	6–10 times	71	19%
	>10 times	221	59%
		Mean	SD
Quality of care (1–4)			
	Encounter	3.58	0.61
	Participation: empowerment	3.12	0.78
	Participation: information	3.05	0.78
	Support	3.27	0.82
	Discharge	2.84	0.84
	Environment	3.38	0.66
	Next of kin	3.23	0.94
	Accessibility	2.82	0.83
Patient-staff interaction (1–4)			
	Establish relationship	3.50	0.62
	Showing interest	2.95	0.77
	Establish structure	2.24	0.94
Mental health outcomes			
Symptom severity (1–5)		2.48	0.76
Recovery (1–5)		3.33	0.71
Quality of life (1–7)		4.17	0.94

a Patients can have more than one diagnosis or psychiatric treatment. Thus, the per cent will not sum up to 100%.

b If the individual is between the ages of 19 and 64 and will never be able to work, now or in the future, due to sickness or a disability.

c If the individual is between 19 and 29 and cannot work for at least 1 year due to sickness or a disability.

d Individuals can apply for a retirement pension from the month they become 62 at the earliest.

### 2.2 Measurements

#### 2.2.1 Quality of life

The Swedish version (32) of the *Manchester Short Assessment of Quality of Life* (MANSA (33)) was used to assess the perceived QoL. The MANSA contains 12 items on global life satisfaction, job, financial situation, friendships, leisure activities, accommodation, personal safety, people that the person lives with, family and health. Items are scored on a 7-point scale from 1 (could not be worse) to 7 (could not be better). A higher score indicates perceived better QoL. The Swedish version has a satisfactory internal consistency with a Cronbach’s alpha of 0.81 (32).

#### 2.2.2 Mental health service provision

The *Quality in Psychiatric Care – Outpatient* (QPC-OP (34)) instrument was used to assess patients’ perception of the quality of care. The QPC-OP consists of 30 items covering 8 dimensions: encounter (6 items), participation-empowerment (3 items), participation-information (5 items), discharge (3 items), support (4 items), environment (3 items), next of kin (2 items), and accessibility (4 items). Each item begins with the wording “I experience that…” and is scored on a 4-point Likert-type scale from 1 (totally disagree) to 4 (totally agree) with a ‘not applicable option.’ A higher score represents perceived better quality of care. The QP-OP has an excellent internal consistency with a Cronbach’s alpha of 0.95 (34).

The *Verbal and Social Interaction questionnaire for Psychiatric Outpatient Care* (VSI-OP (35)) was used to assess the patients’ perceptions of the patient-staff relationship. The VSI-OP contains 17 items covering 3 dimensions: relationship (inviting the patient to establish a relationship, 6 items), interest (showing interest in the patients’ feelings, experiences, and behaviour, 6 items) and helping (helping the patients to establish structure and routines in their everyday life, 5 items). The items are scored on 4-point Likert-type scales from 1 (not at all) to 4 (very high degree). A higher score reflects a perceived better patient-staff relationship. The internal consistency for VSI-OP is satisfactory, with a Cronbach’s alpha of 0.81.

*Psychiatric treatment*. Patients were asked to report which psychiatric treatments they received from the outpatient clinic, e.g., pharmacological, electroconvulsive therapy, counselling, psychotherapy, and other psychiatric treatment. Patients could report having more than one treatment.

#### 2.2.3 Mental health status

The *Symptom Checklist 9 short index* (SCL-9S (36)) was used to measure general psychological distress of patients. The SCL-9S is a unidimensional measure that comprises the nine items most indicative of each of the nine subscales of the Symptom Checklist-90-R (37). Each item is scored on a five-point Likert-type scale from 1 (not at all) to 5 (very much). The internal consistency for SCL-9S is satisfactory, with a Cronbach’s alpha of 0.75 (36).

*Questionnaire about the Process of Recovery* (QPR). The Swedish 16-item one-factor version (28) of the original 22-item two-factor version (38) was used. Each item is scored using a five-point Likert-type scale from 1 (disagree strongly) to 5 (agree strongly). The Cronbach’s alpha internal consistency of the QPR was excellent, with a Cronbach’s alpha of 0.92 (28).

#### 2.2.4 Sociodemographic characteristics

The following sociodemographic variables were used: age, gender, living with partner, education level, and occupation/income source.

#### 2.2.5 Self-reported clinical diagnoses

The patients reported their diagnoses in free text on the questionnaire. The diagnoses were then categorised into eight categories: anxiety, bipolar, dependency disorder, depression, eating disorders, neuropsychological disorders, personality disorders, and schizophrenia. Patients could report having more than one diagnosis.

### 2.3 Data analysis

IBM SPSS 27 and AMOS 25.0 were used to analyse the data. The 333 questionnaires with 15% or more missing items were discarded prior to analysis. Imputation of missing values was performed for the remaining 373 questionnaires using the SPSS expectation-maximisation procedure. We used structural equation modelling to test the proposed model because of greater flexibility in model specification and estimation options, such as simultaneously testing the full model (24, 39). To examine direct and indirect associations we used bootstrapping because it does not make assumptions about the distribution of the variables, which circumvents deviations from multivariate normality (39).

Based on the conceptual model (Figure 1), we specified a testable model and analysed it stepwise. First, we tested the model for the association of variables in mental health care service provision with mental health status variables and QoL. We used an iterative process by deleting non-significant variables and modifying the model according to the modification indices until no more improvements could be made. Once the relationships were established, sociodemographic variables and clinical diagnoses were entered into the model to evaluate whether they still hold when adjusted for these variables.

Model adequacy was assessed with different fit indexes, including the chi-square test, the comparative fit index (CFI), the standardised root mean square residual (SRMR), and the root mean square error of approximation (RMSEA). A non-significant chi-square reflects agreement between the model and the data. CFI values ≥0.90 and ≥0.95 and SRMR and RMSEA values ≤0.08 and ≤0.05 were considered adequate and excellent levels of goodness of fit, respectively (23).

Differences between the goodness-of-fit indexes of the models were analysed to determine which model better fitted the data. Differences no greater than 0.01 between the CFI values (0.030 for the SRMR and 0.015 between the RMSEA values) were considered irrelevant when comparing the models (40). In such cases we chose the model that accomplished the desired level of explanation with as few parameters or predictor variables as possible.

## 3 Results

Twenty significant relationships were found after the iterative testing procedure (Table 2), and we reached a final mediation model (Table 3) that received excellent goodness of fit for all evaluated goodness-of-fit indices. The non-significant chi-square shows that the model did not deviate significantly from observed data. The *R*^2^ indicates that the model explained 55% of the variance in quality of life, which was deemed adequate. Adjustment of the model with sociodemographic and diagnosis variables had a small and non-significant effect on the model fit (Δ*χ*^2^ = 17.04 with a Δ*df* = 21 giving a *p* = 0.71, a ΔRMSEA = 0.011, and a ΔCFI = 0.001), demonstrating that the associations between the variables in mental health service provision and QoL were not affected by the observed socioeconomic or diagnosis variables.

**Table 2:** Standardised regression weights.

Predictor		Estimate	
Mental health service provision variables			
QPC Access	➔	QPC Discharge	0.223***
QPC Next of kin	➔	QPC Discharge	0.181***
QPC Support	➔	QPC Discharge	0.122*
QPC Participation empowerment	➔	QPC Discharge	0.154**
QPC Participation information	➔	QPC Discharge	0.259***
QPC Participation information	➔	Quality of life (MANSA)	0.062*
QPC Discharge	➔	Symptom severity (SCL-9S)	−0.117*
QPC Discharge	➔	Recovery (QPR)	0.204***
QPC Environment	➔	Symptom severity (SCL-9S)	−0.143**
VSI Relationship	➔	Recovery (QPR)	0.099*
Treatment: Psychotherapy	➔	Symptom severity (SCL-9S)	0.169***
Mental health status variables			
Symptom severity (SCL-9S)	➔	Recovery (QPR)	−0.529***
Symptom severity (SCL-9S)	➔	Quality of life (MANSA)	−0.280***
Recovery (QPR)	➔	Quality of life (MANSA)	0.499***
Sociodemographic and diagnosis variables			
Living	➔	Quality of life (MANSA)	0.129***
Bipolar disorder	➔	Quality of life (MANSA)	0.122***
Schizophrenia	➔	Symptom severity (SCL-9S)	−0.101*
Schizophrenia	➔	Quality of life (MANSA)	0.087*
Anxiety	➔	Symptom severity (SCL-9S)	0.176***
Anxiety	➔	Treatment: Psychotherapy	0.201***

Parameter estimate coefficients are standardised. QPC, The quality in psychiatric care – outpatient questionnaire; VSI, the verbal and social interaction questionnaire; SCL-9S, symptom checklist 9 short index; QPR, questionnaire about the process of recovery. *p < 0.05, **p < 0.01, and ***p < 0.001.

**Table 3:** Final mediation model of direct, indirect, and total effects on quality of life.

	Direct	Indirect	Total
Mental health service provision variables			
QPC Access		0.037***	0.037***
QPC Next of kin		0.030***	0.030***
QPC Support		0.020*	0.020*
QPC Environment		0.078**	0.078**
QPC Participation empowerment		0.025**	0.025**
QPC Discharge		0.165***	0.165***
QPC Participation information	0.062*	0.043*	0.105**
VSI Relationship		0.049*	0.049*
Treatment: Psychotherapy		−0.092***	−0.092***
Mental health status variables			
Symptom severity (SCL-9S)	−0.280***	−0.264^***^	−0.544***
Recovery (QPR)	0.499***		0.499***
Sociodemographic and diagnosis variables			
Living	0.129***		0.129***
Anxiety		−0.114^***^	−0.114***
Bipolar disorder	0.122***		0.122***
Schizophrenia	0.087**	0.073^**^	0.160**
Model fit			
Chi-square; degree of freedom; value of p	*χ*^2^ = 49.502; *df* = 45; *p* = 0.230		
RMSEA (90% CI)	0.020 (0.001; 0.042)		
CFI	0.997		
SRMR	0.024		
Model *R*^2^	0.545		

Parameter estimate coefficients are standardised. RMSEA, root mean square error of approximation; CFI, comparative fit index; SRMR, standardised root mean squared residual; QPC, The quality in psychiatric care – outpatient questionnaire; VSI, the verbal and social interaction questionnaire; SCL-9S, symptom checklist 9 short index; QPR, questionnaire about the process of recovery. *p < 0.05, **p < 0.01, and ***p < 0.001.

**Figure 2:**
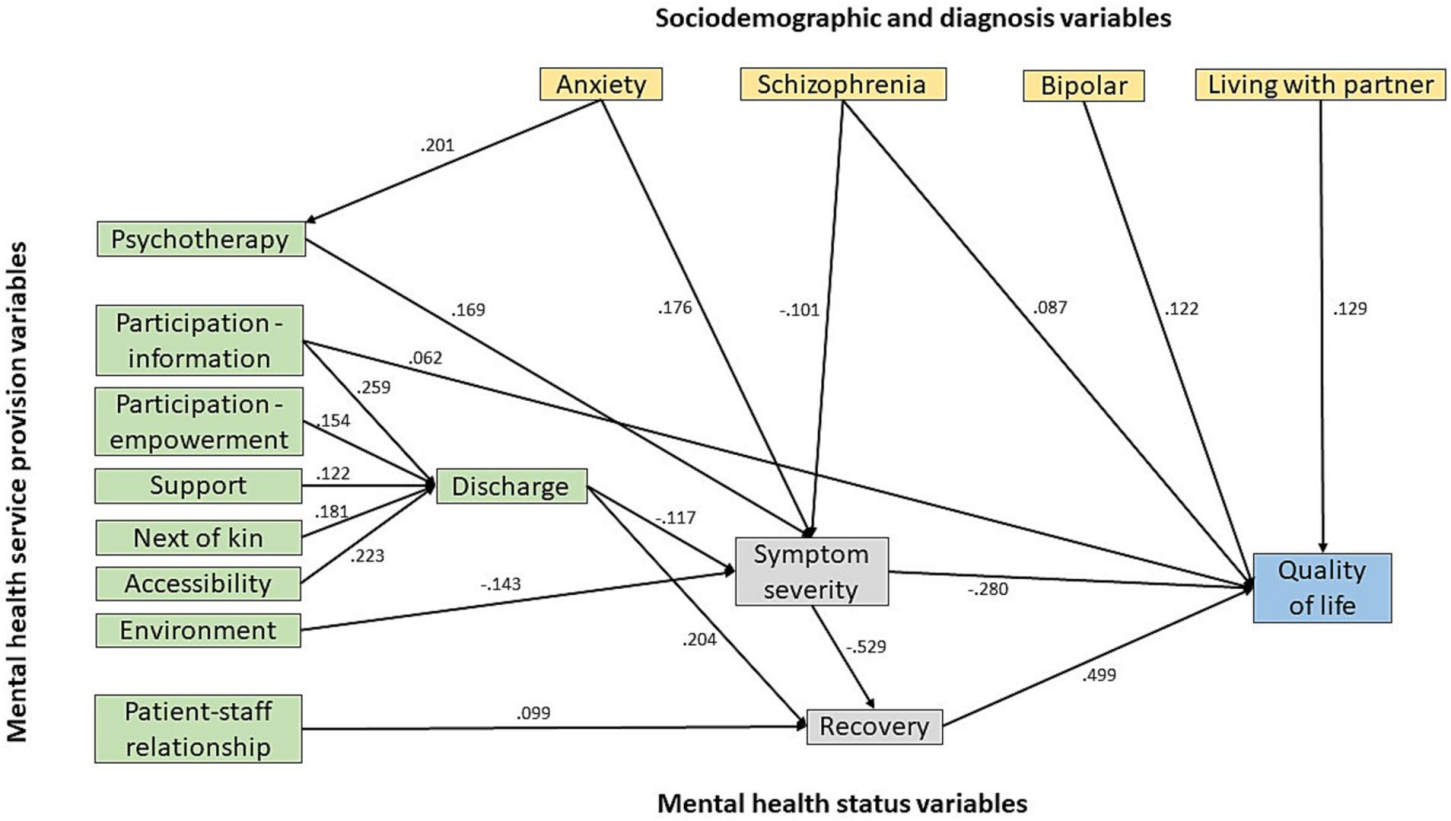
Final mediation model of the impact of mental health service provision on psychiatric outpatients’ perception of quality of life. Coefficients are standardised.

The final model indicated that the relationship between the variables in mental health service provision and the patient’s perceived QoL is relatively complex. As shown in Figure 2, QoL was directly associated with six variables, whereas only one, participation information, was a service provision variable. Most service provision variables were mediated by the mental health status variables (symptom severity and recovery) mediating QoL. Among the quality-of-care variables, all but one were mediated by the discharge variable. Moreover, the patient-staff relationship, measured by the VSI, had an indirect association with QoL and a significant direct association with recovery. One treatment variable, psychotherapy, had a significant association with QoL, i.e., a mediated association via symptom severity and recovery, resulting in a total negative association with QoL.

Although sociodemographic and diagnosis variables had no significant effects on the model fit, some relationships with QoL were significant. Living with partner had a significant and direct positive association with QoL. For clinical diagnosis, bipolar disorder, schizophrenia, and anxiety had a significant positive association with QoL. Notably, the relationship between patients’ anxiety and QoL was somewhat complex. Patients with anxiety were more likely than those with other diagnoses to receive psychotherapy. Those receiving psychotherapy (whether or not with anxiety) perceived slightly more severe symptoms and less QoL than those receiving other psychiatric treatments, resulting in a negative association between anxiety and QoL. In contrast, patients diagnosed with schizophrenia and bipolar disorders reported better QoL than patients with other diagnoses.

## 4 Discussion

This study examined the relationship between perceived mental health service provision and QoL among patients in psychiatric outpatient care. The final model achieved excellent goodness of fit, showing that variables in mental health service provision mainly had an indirect effect on patients’ perceived QoL and that this effect was mediated by symptom severity and recovery.

This result is consistent with research indicating that service provision variables have a minor direct association with perceived QoL, suggesting that other factors beyond mental health care service are more impactful (5, 31, 40). Moreover, the results correspond to studies using complex mediator models. For instance, Fleury and colleagues (21) found that sociodemographic variables (gender and living) and clinical diagnosis variables (mood disorders and substance use disorders) were directly related to patients’ perceived QoL but that only two of the service provision variables (service continuity and adequacy of help) were directly associated with QoL. Similarly, the present study shows that recovery serves as an important mediator but that symptom severity, which was not assessed by Fleury and colleagues (21), also acts as a mediator and has a similar magnitude of association with QoL, suggesting that both recovery and symptom severity have a vital role for patients’ perceived QoL. However, in this study the association of symptom severity with QoL is primarily mediated by recovery, which correspond with findings by Saavedra and colleagues (24), suggesting that the symptom severity and QoL association may depend on the presence of recovery, which aligns with previous research (e.g., (30)). The present results thus suggest that symptom relief is unnecessary for recovery, but in relation to QoL, the absence of symptom relief will reduce recovery, resulting in a negative association with QoL. Hence, the model indicates that recovery would have less impact on patients’ perceived QoL if there was no symptom relief.

Surprisingly, the discharge dimension was a mediator between most of the other service provision variables and recovery. It is not apparent why discharge has this role. However, visits to outpatient clinics are often intermittent and a regular discharge procedure does not always occur. Thus, the QPC-OP discharge dimension items are mainly about closure and the future. These aspects can be related to findings by Fleury and colleagues (21), who showed that recovery, which included elements found in patients’ experience at discharge, was a mediating factor between service continuity and QoL. Discharge may thus be a significant event and a crucial component of the treatment process. As such, discharge is a creative process that can be customised to meet each patient’s unique needs and comprehensively address them across multiple health systems in a continuous and coordinated manner (41). As mentioned above, the discharge process in outpatient care is less formal, and in this study, it was measured by questions including aspects of closure and the future. These questions encompass parts of the personal agency, an essential factor in understanding the ‘service provision-recovery-quality of life’ relationship (22).

From a clinical perspective, this finding raises questions about whether interventions in outpatient psychiatric care need to focus more on agency rather than providing more support and that it is important to include questions about closure and the future from the start of the care process. Prospects encouraging hope can be positive for perceived QoL (42). The quality-of-service provision may also increase patients’ capacity, allowing them to influence their future (43), potentially affecting their QoL. Our model aligns with the assumption that the experience of controlling and being able to master situations is an important recovery factor for developing a higher level of QoL. In this regard, our findings correspond with a holistic perspective on mental health service providers aiming to support recovery by focusing on both symptom reduction and increased well-being related to hope, self-esteem, social connectedness, and a sense of control of one’s life (35–47). However, the current outcome on the role of the discharge dimension should be taken with caution until it has been replicated in independent studies. Further research is needed to clarify the link between the quality of the service provided and the patients’ view of their future.

In addition to the discharge issues, the present results show that the patient-staff relationship, as measured by the VSI, may have an independent effect on recovery. Among the three elements of the patient-staff relationship measured by the VSI-OP, only *Inviting the patient to establish a relationship* had an association with QoL. This association was mediated entirely by recovery. In other words, the greater the patient-staff relationship, the greater the recovery and thus better QoL. A good patient-staff relationship has been noted as an important factor that can make a difference in recovery during an episode of mental illness, including involuntary admission (48). Using the VSI-OP, Rask and colleagues (35) showed that patients reported that *Inviting the patient to establish a relationship*, as well as, *Showing interest in patients’ feelings, experiences, and behaviour* were rated as the most frequent actions performed by staff. In another study, using VSI for supported housing, the residents rated *inviting the patient to establish a relationship* as the most frequently performed and the most important facet of the patient-staff interaction (49). These findings confirm Green et al. (50), who reported that patients value a staff perceived as competent, caring, trustworthy, and trusting and that these factors were important for patient recovery. This position is in line with our findings that a positive patient-staff relationship, including staff showing interest in the patients’ thoughts and experiences, is vital for patient recovery.

Concerning clinical diagnoses, schizophrenia and bipolar disorders were directly associated with QoL. The associations were positive, indicating that patients with schizophrenia and bipolar disorder perceived QoL better than patients with other mental diagnoses. In addition, schizophrenia was associated with less symptom severity, leading to a positive indirect association with QoL. This observation aligns with previous findings showing that patients with schizophrenia report higher self-reported QoL than patients with other mental disorders (51). However, our finding differed from the finding of Priebe et al. (51) that schizophrenia is associated with more severe reported symptoms.

Anxiety was not directly associated with QoL. Instead, the association between anxiety and QoL was mediated by psychotherapy and reported symptom severity, indicating that the relationship between anxiety and QoL is complex. Specifically, patients with anxiety reported receiving more psychotherapy compared to patients with other mental diagnoses and receiving psychotherapy was associated with reporting greater symptom severity, suggesting that patients with anxiety in ongoing psychotherapy also struggle with severe symptoms. This circumstance may thus explain why psychotherapy was associated with greater reported symptom severity.

Concerning the sociodemographic variables only one, living with a partner, was positively associated with QoL. Although associations between sociodemographic variables and QoL seem to depend on the study population, similar results have been demonstrated in studies on patients with schizophrenia (52).

### 4.1 Methodological considerations

The measurement of QoL in mental health services depends on the assessment instrument used. We chose the MANSA because it is an established and widely used instrument for QoL assessment in mental health practice and has good psychometric properties in Swedish patients who receive psychiatric outpatient services. Because QoL instruments may differ in definition and item content, results based on other tools may deviate from those we observed. Yet, our results are largely consistent with comparable results in studies using other QoL measures (e.g., (20, 21, 26)). In addition, the rigorous method of structural equation modelling made it possible to investigate the complex network of mediating variables and to model associations and hypothesised causal mechanisms between service provision and QoL.

This study has some limitations. First, the model hinges on the study variables. We have chosen variables based on previous research, which does not rule out the possibility that other variables may have equal or better predictive values than those we chose. Second, as in any research involving recall, there exists the potential for the results to be influenced by recall bias. Nevertheless, in our study, patients complete the questionnaire immediately upon leaving the clinic. The short duration between their visit/consultation and answering the questionnaire significantly reduces the likelihood of substantial recall bias affecting the outcomes. Third, although the study included patients from several clinics in three regions in Sweden, the results may not be directly generalisable to other countries, especially those with different healthcare systems. Fourth, because the present data are cross-sectional, we cannot determine the temporal and causal relationships of the variables. This model must therefore be assessed in terms of the model matching the observed data, given that the model reflects an existing temporal/causal relationship. Thus, the results do not reflect actual causal relationships. Therefore, longitudinal or experimental studies should be performed to investigate the causality of the effects of mental health service provision on patients’ QoL. In such an effort, the present study can thus be used to scrutinise potential causal relationships more closely.

## 5 Conclusion

The present study shows that patients’ perception of mental health service provision is positively associated with their perceived QoL; however, this association is mostly indirect and mediated by reduced symptom severity and increased recovery. This finding can help design future interventions to enhance service provision and thus promote patients’ QoL. Further studies are needed to capture a causal path to QoL.

## Data availability statement

The datasets presented in this article are not readily available because of ethical restrictions. Requests to access the datasets should be directed to the corresponding author.

## Ethics statement

The studies involving humans were approved by the Swedish Ethical Review Authority. The studies were conducted in accordance with the local legislation and institutional requirements. The ethics committee/institutional review board waived the requirement of written informed consent for participation from the participants or the participants’ legal guardians/next of kin because the participants gave verbal informed consent to participate.

## Author contributions

L-OL: Conceptualization, Formal analysis, Methodology, Writing – original draft, Writing – review & editing. PR: Conceptualization, Writing – review & editing. MR: Conceptualization, Writing – review & editing. DB: Conceptualization, Writing – review & editing. TS: Conceptualization, Writing – review & editing. KG: Conceptualization, Writing – review & editing. IR: Conceptualization, Writing – review & editing. AS: Conceptualization, Funding acquisition, Writing – review & editing.
